# Effect of body position changes on endotracheal tube cuff pressure in ventilated patients

**DOI:** 10.1186/cc12092

**Published:** 2013-03-19

**Authors:** C Lizy, W Swinnen, S Labeau, S Blot

**Affiliations:** 1 Gent University, Ghent, Belgium; 2 General Hospital Sint Blasius, Dendermonde, Belgium; 3 University College Ghent, Belgium

## Introduction

In order to avoid either microaspiration (<20 cmH_2_O) or tracheal injury (>30 cmH_2_O), the target for endotracheal tube (ETT) cuff pressure (CP) is between 20 and 30 cmH_2_O. Our objective was to assess the effect of patient positioning changes on CP in adult patients.

## Methods

A sample of 12 orally intubated and sedated patients was selected. Patients received neuromuscular blockade and were positioned in a neutral start position (backrest, head of bed elevation (HoB) 30°, head in neutral position) and CP set at 25 cmH_2_O. Subsequently 16 different position changes were performed: anteflexion head, hyperextension head, left and right lateral flexion of head, left and right rotation of the head, semirecumbent position (HoB 45°), recumbent position (HoB 10°), horizontal position, trendelenburg (-10°), left and right lateral positioning over 30°, 45° and 90°. Once the patient was correctly positioned, CP was recorded during an end-expiratory ventilatory hold. CP observed was compared with CP at start position (25 cmH_2_O). Also the number of values outside the target range (20 to 30 cmH_2_O) was reported as considered clinically relevant.

## Results

A total of 192 measurements were performed (12 subjects×16 positions). Results are shown in Table 1 (CP reported as median, IQR). In every position a significant deviation in CP was observed. In total, 40.6% of values exceeded the upper limit of 30 cmH_2_O. No values beneath the lower target limit of 20 cmH_2_O were observed. In each position the upper target limit was exceeded at least once.

**Table 1:** Endotracheal tube cuff pressure values following patient position changes

	Cuff pressure (cmH_2_O)^a^	*P *for Δ from start position (25 cmH_2_O)	Measurements >30 cmH_2_O (*n *(%))
Anteflexion head	33.5 (27.5 to 42.0)	0.005	9 (75)
Hyperextension head	30.5 (27.5 to 42.0)	0.004	6 (50)
Lateral flexion head, left	26.5 (25.3 to 28.8)	0.025	1 (8)
Lateral flexion head, right	27.0 (25.3 to 29.8)	0.049	2 (17)
Rotation head, left	27.0 (26.3 to 33.8)	0.003	3 (25)
Rotation head, right	28.0 (26.3 to 38.8)	0.02	4 (33)
Semirecumbent, 45° HoB	28.0 (25.3 to 31.5)	0.009	2 (17)
Recumbent, 10° HoB	28.0 (25.3 to 31.5)	0.016	3 (25)
Horizontal back rest	28.5 (25.3 to 31.5)	0.011	3 (25)
Trendelenburg, 10°	29.0 (25.3 to 32.0)	0.008	5 (42)
Left lateral position, 30°	30.5 (25.3 to 32.0)	0.01	6 (50)
Left lateral position, 45°	30.5 (27.3 to 32.8)	0.005	6 (50)
Left lateral position, 90°	34.0 (27.0 to 36.8)	0.006	7 (58)
Right lateral position, 30°	31.0 (27.0 to 34.5)	0.003	6 (50)
Right lateral position, 45°	32.5 (28.3 to 35.8)	0.002	8 (50)
Right lateral position, 90°	31.0 (29.3 to 35.0)	0.003	7 (58)
Total (192 measurements)	-	-	78 (40.6)

HoB, head of bed elevation. ^a^Cuff pressure values reported as median (first to third quartile).

## Conclusion

Simple changes in patient positioning may result in potentially harmful CP (>30 cmH_2_O). These observations call for strict CP monitoring.

